# Age-Stratified Transcriptomic Profiling Identifies CEMIP as a Candidate Biomarker in Early-Onset Colorectal Cancer and Reveals an Association with PTCH1-Related Hedgehog Signaling

**DOI:** 10.3390/genes17080922

**Published:** 2026-08-04

**Authors:** Chung-Ying Lee, Hsu-Jui Pan, Yu-Cheng Lee, Hieu Duc Nguyen, Yi-Chun Ni, Ke Xin Yee, Man Thi Nguyen, Yung-Fu Wu, Kuen-Haur Lee

**Affiliations:** 1 Division of Gastroenterology and Hepatology, Department of Internal Medicine, Taipei Medical University Shuang Ho Hospital Ministry of Health and Welfare, New Taipei City 23561, Taiwan; 12310@s.tmu.edu.tw; 2 Division of Gastroenterology and Hepatology, Taipei Medical University School of Medicine, Taipei 11031, Taiwan; 3 Graduate Institute of Cancer Molecular Biology and Drug Discovery, College of Medical Science and Technology, Taipei Medical University-Shuangho Campus, New Taipei City 23561, Taiwan; panray8802069487@gmail.com (H.-J.P.); mannguyenthi2001@gmail.com (M.T.N.); 4 Graduate Institute of Medical Sciences, College of Medicine, Taipei Medical University, Taipei 11031, Taiwan; yclee0212@tmu.edu.tw; 5 PhD Program for Cancer Molecular Biology and Drug Discovery, College of Medical Science and Technology, Taipei Medical University-Shuangho Campus, New Taipei City 235, Taiwan; hieudn206@gmail.com (H.D.N.); ckni1012@gmail.com (Y.-C.N.); d621112004@tmu.edu.tw (K.X.Y.); 6 Department of Medical Research, Tri-Service General Hospital, National Defense Medical University, Taipei 114, Taiwan; 7 Graduate Institute of Medical Sciences, College of Medicine, National Defense Medical University, Taipei 114, Taiwan; 8 TMU Research Center for Digestive Medicine, Taipei Medical University, Taipei 110, Taiwan; 9 Cancer Center, Wan Fang Hospital, Taipei Medical University, Taipei 110, Taiwan; 10 TMU Research Center of Cancer Translational Medicine, Taipei Medical University, Taipei 110, Taiwan

**Keywords:** early-onset colorectal cancer, CEMIP, Hedgehog signaling, transcriptomics

## Abstract

Background/Objectives: Early-onset colorectal cancer (EOCRC), defined as colorectal cancer diagnosed before 50 years of age, is increasing worldwide and may exhibit molecular features distinct from late-onset colorectal cancer (LOCRC). This study aimed to identify EOCRC-associated genes and evaluate the functional relevance of the leading candidate. Methods: Public transcriptomic datasets were used for age-stratified candidate-gene discovery and validation. Overall survival analysis prioritized candidates, followed by loss-of-function studies and pathway-focused analyses in colorectal cancer cell lines. Results: CEMIP, NKD2, and FOXQ1 were elevated in the EOCRC groups in the discovery and integrated validation analyses. Among them, only CEMIP was significantly associated with poorer overall survival. HCT116 and HT29 cells showed relatively high endogenous CEMIP expression, and CEMIP knockdown reduced relative viable cell biomass and migration in wound-healing and Transwell assays. CEMIP-associated transcripts were enriched in several pathways, including Hedgehog, Wnt/β-catenin, and TGF-β signaling. In TCGA colon adenocarcinoma samples, CEMIP correlated most strongly with PTCH1; however, the correlations with other Hedgehog components were weak or absent. CEMIP silencing decreased PTCH1 and GLI1 transcripts while increasing SMO, suggesting a coordinated but non-linear relationship. Conclusions: CEMIP is an EOCRC-associated candidate biomarker and a functional contributor to colorectal cancer cell viability and migration. Its relationship with PTCH1-related Hedgehog signaling is exploratory and warrants direct mechanistic validation.

## 1. Introduction

Colorectal cancer (CRC) is the third most common malignancy and the second leading cause of cancer-related death worldwide, accounting for approximately 1.9 million new cases and 900,000 deaths in 2020 [1]. More than 90% of CRCs are adenocarcinomas arising from the glandular epithelium of the colon and rectum [2]. Most CRC cases are sporadic and develop through multistep tumorigenic processes involving adenomatous or serrated precursor lesions, including the adenoma-carcinoma sequence, the serrated pathway, and, less commonly, inflammation-associated tumorigenesis [3,4,5,6,7,8]. Although the incidence and mortality of CRC in older adults have gradually declined owing to screening, early detection, and treatment advances [9], the incidence of early-onset colorectal cancer (EOCRC), generally defined as CRC diagnosed before the age of 50 years, has continued to increase over the past two decades [10,11,12,13,14,15]. A large European cohort study further showed that CRC incidence increased annually by 7.9% in individuals in their 20s, 4.9% in those in their 30s, and 1.6% in those in their 40s, underscoring the growing clinical importance of EOCRC [16,17].

EOCRC is increasingly recognized as a distinct and heterogeneous disease entity. Although some cases are attributable to hereditary cancer syndromes or familial predisposition, more than 80% of patients with EOCRC do not fall into these categories [10,11]. Compared with late-onset colorectal cancer (LOCRC), EOCRC is more frequently diagnosed at an advanced stage and is often associated with more aggressive clinicopathological features [18,19]. These observations suggest that EOCRC may harbor distinct molecular characteristics; however, no unified molecular mechanism or specific biomarker has yet been established [9]. Because tumor metastasis is a major cause of cancer-related mortality, pathways involved in epithelial–mesenchymal transition (EMT), invasion, and dissemination are of particular interest in EOCRC [20]. EMT is regulated by several oncogenic pathways, including TGF-β, Wnt, and Hedgehog signaling [21,22,23,24]. Among these, the Hedgehog (Hh) signaling pathway is an evolutionarily conserved signaling cascade involved in embryonic development, tissue maintenance, and tumorigenesis [25,26]. Canonical Hh signaling is activated when Hedgehog ligands bind to Patched1 (PTCH1), thereby relieving Smoothened (SMO) inhibition and inducing downstream GLI family zinc finger 1 (GLI1)-dependent transcription [27,28,29]. Dysregulated Hh signaling has been associated with tumor growth, invasion, and malignant progression in multiple cancers [30,31], suggesting that it may also contribute to the aggressive behavior of EOCRC.

Several genes implicated in tumor progression may be relevant to EOCRC biology. Cell migration-inducing protein (CEMIP), also known as KIAA1199 or HYBID, has been linked to hyaluronic acid depolymerization, tumor progression, epithelial–mesenchymal transition (EMT), and metastasis [32]. CEMIP is upregulated during the transition from normal colorectal epithelium to adenomatous and neoplastic lesions and remains elevated in colorectal adenocarcinoma [33,34,35]. NKD2, a negative regulator of Wnt signaling, and FOXQ1, a Forkhead box transcription factor associated with EMT, invasion, and metastasis, have also been implicated in CRC [36,37,38,39,40,41,42,43,44,45,46,47,48,49,50]. However, whether these genes are enriched in age-defined EOCRC cohorts remains unclear. We therefore integrated public transcriptomic datasets to identify EOCRC-associated candidates and used survival and in vitro loss-of-function analyses to prioritize and characterize CEMIP. Because pathway enrichment suggested Hedgehog signaling, we additionally examined the association of CEMIP with PTCH1, SMO, and GLI1. This pathway analysis was designed as hypothesis-generating rather than as evidence of a direct molecular interaction.

## 2. Materials and Methods

### 2.1. Public Dataset Analysis

Publicly available gene-expression datasets were retrieved from the NCBI Gene Expression Omnibus (GEO). GSE21815 was used as the discovery cohort, with cases stratified as EOCRC (<50 years) or LOCRC (≥50 years) according to the deposited age-at-diagnosis metadata. Ten previously identified CRC-associated genes were compared between the two groups. Integrated validation was performed using GSE20970, GSE36335, GSE37892, GSE45404, GSE56386, GSE73360, GSE89076, GSE101896, GSE106582, and GSE161158. For pathway analysis, GSE39575, comprising three control and three CEMIP-overexpressing SW480 samples, was analyzed; genes with nominal *p* < 0.05 were submitted for enrichment analysis. Detailed histological grade, complete TNM categories, molecular subtype, and treatment variables were not consistently available across the deposited metadata and were therefore not used for clinicopathological subgroup analyses.

### 2.2. Survival and Gene-Correlation Analyses

Overall survival associations for NKD2, FOXQ1, and CEMIP were assessed using Kaplan–Meier Plotter in the colorectal cancer cohort (Department of Bioinformatics, Semmelweis University, Budapest, Hungary; accessed in June 2026) [51]. Patients were separated into high- and low-expression groups using the plat-form-defined optimal cutoff, and hazard ratios (HRs), 95% confidence intervals (CIs), and log-rank *p* values were recorded. Gene-expression correlations between CEMIP and PTCH1, SMO, or GLI1 were evaluated using ENCORI/starBase in The Cancer Genome Atlas colon adenocarcinoma (TCGA-COAD) cohort (*n* = 471) [52]. The coefficients and *p* values generated by the platform were reported. Because complete individual-level clinicopathological and molecular covariates were not available through these interfaces for all analyzed cases, multivariable and age-specific survival subgroup analyses were not performed.

### 2.3. Enrichment Analysis and Visualization

Genes associated with CEMIP overexpression in GSE39575 were analyzed using Enrichr against the MSigDB Hallmark 2020 collection. Enrichment results were visualized using ImageGP. Nominal *p* values and multiple-testing-adjusted *p* values were retained, and the complete output is provided in Supplementary Table S1. Pathways were considered hypothesis-generating and were not interpreted as evidence of direct protein–protein interaction or pathway dependence.

### 2.4. Cell Lines, Culture Conditions, and Reagents

The selection of colorectal cancer cell lines was guided by the age of the patients from whom the original tumors were derived, based on the molecular characterization reported by Medico et al. [53]. Among the colorectal cancer cell lines with available patient-age information, HCT116 and HT29 were derived from patients younger than 50 years and were therefore used as EOCRC-derived models, whereas HCT15 and WiDr were derived from patients aged 50 years or older and were used as LOCRC-derived models. CRL-1459 was included as a non-malignant colon-derived reference cell line. HCT116 and HT29 were further selected for downstream functional analyses because they exhibited relatively high endogenous CEMIP expression. Cells were cultured in RPMI-1640 medium (Corning Incorporated, Corning, NY, USA) supplemented with 10% fetal bovine serum (FBS) (Corning Incorporated, Corning, NY, USA) and 1% penicillin-streptomycin (Thermo Fisher Scientific, Waltham, MA, USA; final concentrations: 50 U/mL penicillin and 50 μg/mL streptomycin). Cells were maintained at 37 °C in a humidified incubator containing 5% CO_2_.

### 2.5. Cell Transfection

For loss-of-function experiments, a CEMIP-targeting siRNA and a general non-targeting negative-control siRNA were purchased from Invitrogen (Carlsbad, CA, USA). The control siRNA was not designed as a sequence-matched control specifically for the CEMIP-targeting siRNA; it was used to account for nonspecific effects of oligonucleotide delivery and transfection. The exact siRNA sequences were not available in the archived experimental records and therefore are not reported. HCT116 and HT29 cells were transfected with 50 or 100 nM siRNA using jetPRIME transfection reagent (Polyplus-transfection, New York, NY, USA) according to the manufacturer’s instructions. Knockdown efficiency was assessed at the mRNA level by RT-qPCR 24 h after transfection.

### 2.6. Quantitative Reverse-Transcription PCR (RT-qPCR)

Total RNA was extracted using the RNeasy Kit and QIAshredder columns (Qiagen, Valencia, CA, USA), and 1 μg of RNA was reverse-transcribed using the iScript cDNA Synthesis Kit (Bio-Rad, Hercules, CA, USA). Quantitative PCR was performed with Qiagen SYBR Green reagent using the following program: 95 °C for 3 min; 45 cycles of 95 °C for 5 s, 55 °C for 20 s, and 72 °C for 5 s; followed by melt-curve analysis. Each sample was analyzed in triplicate. Transcript abundance was quantified using the standard-curve method and normalized to 18S rRNA. The primer sequences (5′–3′) used in this study were as follows: CEMIP, forward GCTCTTGAGTTGCATGGACA and reverse ACCGCGTTCAAATACTGGAC; NKD2, forward AACACGGAGCGCAGAAAC and reverse GGCC-GAATCTGGACGTGTA; FOXQ1, forward GCAAGTTCCCCTTTTTCC and reverse AGTCGTTGAGCGAAAGGTT; PTCH1, forward TGCTGTGCCTGTGGTCATCCTGATT and reverse CAGAGCGAGCATAGCCCTGTGGTTC; SHH, forward GATGTCTGCTGCTAGTCCTCG and reverse CACCTCTGAGTCATCAGCCTG; SMO, forward TGGCCG-CTTCAGATGACAGATGTTG and reverse CGTTAGCCGAATGTCAGCCGTGAAG; GLI1, forward CCCATAGGGTCTCGGGGGTCTCAAAC and reverse GGAGGACCTGCGGCTGACTGTGTAA; and 18S, forward CATGGCCGTTCTTAGTTGGTGG and reverse CGCTGAGCCAGTCAGTGTAG.

### 2.7. Western Blot Analysis

Basal CEMIP expression was examined in the cell-line panel. Cells were lysed in buffer containing protease and phosphatase inhibitors, and lysates were centrifuged at 12,000 rpm for 10 min. Protein concentrations were determined using a Bio-Rad protein assay with bovine serum albumin as the standard. Equal amounts of protein (30 μg) were separated by SDS-PAGE, transferred to PVDF membranes, blocked with 5% bovine serum albumin, and incubated with primary antibodies overnight at 4 °C. After incubation with secondary antibodies (1:10,000), bands were detected by enhanced chemiluminescence using an Amersham Imager 600 (GE Healthcare Bio-Sciences AB, Uppsala, Sweden) and quantified with ImageJ (National Institutes of Health, Bethesda, MD, USA). Protein-level knockdown was not assessed in the archived experiments.

### 2.8. Cell Viability Assay

Relative viable cell biomass was measured by crystal violet staining. At 24 h after siRNA transfection, cells were seeded in 96-well plates and cultured for an additional 24 h. Cells were stained with 0.5% crystal violet for 10 min, washed, dried, and solubilized in 0.1 M sodium citrate. Absorbance was measured at 550 nm and expressed relative to the control siRNA group. This assay estimates adherent viable cell biomass and was not interpreted as a direct Ki67- or cell-cycle-based proliferation measurement.

### 2.9. Transwell Migration Assay

Cell migration was assessed using Transwell chambers with 8-μm pore-size membranes. At 24 h after control or CEMIP siRNA transfection, 5 × 10^5^ HCT116 or HT29 cells in serum-free medium were seeded in the upper chamber; the lower chamber contained medium supplemented with 10% fetal bovine serum as a chemoattractant. After 12 h, cells that had traversed the membrane and reached its lower surface were fixed with methanol, stained with Giemsa, imaged, and quantified. The blue-purple staining in the representative micrographs identifies migrated Giemsa-positive cells.

### 2.10. Wound-Healing Assay

The effect of CEMIP on cell migration was further assessed using a wound-healing assay kit (ibidi, Gräfelfing, Germany). HCT116 and HT29 cells were seeded at 4 × 10^5^ cells/well. After 24 h, the inserts were removed to generate a wound gap, and images were captured at 0, 8, 12, and 24 h. The wound-healing rate was quantified using ImageJ.

### 2.11. Statistical Analysis

Cell-based experiments were performed in three independent biological replicates and are presented as the mean ± standard deviation. Two-group comparisons were performed using an unpaired two-tailed Student’s *t*-test in GraphPad Prism 8.0. A *p* value < 0.05 was considered statistically significant. Survival analyses used the log-rank test and HRs generated by Kaplan–Meier Plotter. Correlation coefficients and *p* values were obtained from ENCORI. Enrichment results are reported with both nominal and adjusted *p* values; only pathways meeting the database-adjusted significance threshold were described as statistically enriched.

### 2.12. Generative Artificial Intelligence Disclosure

Generative artificial intelligence (GenAI), specifically ChatGPT (OpenAI, GPT-5.6 Thinking), was used only for language refinement and manuscript editing. No GenAI tools were used for data analysis, data interpretation, figure generation, or scientific decision-making.

## 3. Results

### 3.1. Integrated Transcriptomic Analyses Identify CEMIP, NKD2, and FOXQ1 as EOCRC-Associated Candidates

**Figure 1:**
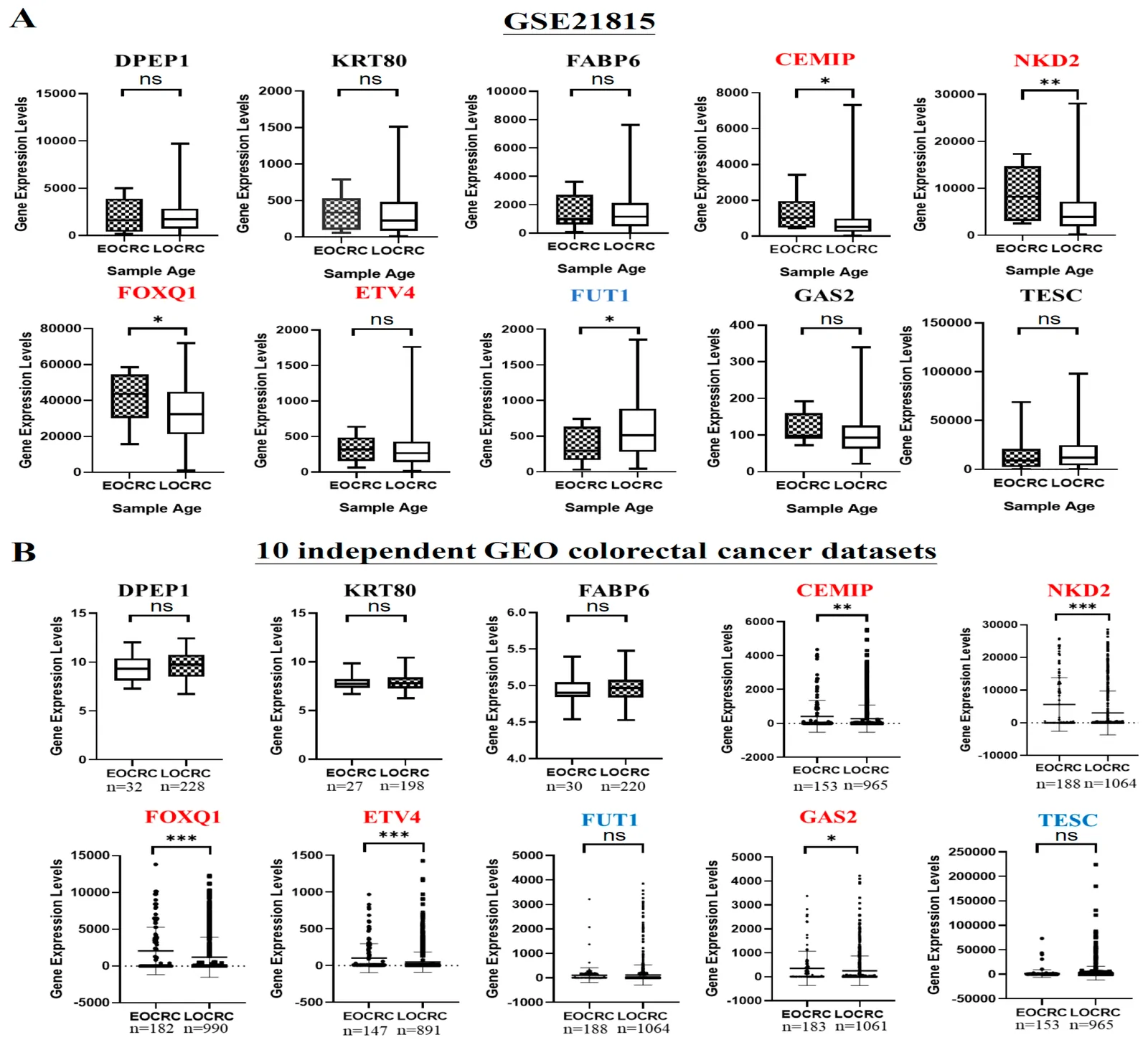
Integrated expression analysis of ten CRC-associated genes in EOCRC and LOCRC. (**A**) Age-stratified expression in the GSE21815 discovery cohort. CEMIP, NKD2, and FOXQ1 were significantly higher in EOCRC. (**B**) Integrated analysis of ten independent GEO colorectal cancer datasets showing the same direction of differential expression for CEMIP, NKD2, and FOXQ1. Red labels indicate genes higher in EOCRC; blue labels indicate genes higher in LOCRC; ns: non-significant. * *p* < 0.05; ** *p* < 0.01; *** *p* < 0.001.

Based on our previous study, ten genes (DPEP1, KRT80, FABP6, NKD2, FOXQ1, CEMIP, ETV4, TESC, FUT1, and GAS2) were selected for age-stratified evaluation [54]. In GSE21815, CEMIP, NKD2, and FOXQ1 were significantly higher in the EOCRC group than in the LOCRC group (Figure 1A). The available clinical characteristics of the discovery cohort are summarized in Table 1. Integrated analysis of ten independent GEO datasets showed the same direction of differential expression for these three genes (Figure 1B). Detailed histological grade, complete TNM categories, molecular subtype, and treatment information were not consistently available in the deposited metadata; therefore, robust clinicopathological correlation analyses could not be performed. These results identify CEMIP, NKD2, and FOXQ1 as candidate genes associated with age-defined EOCRC cohorts.

### 3.2. Overall Survival Analysis Prioritizes CEMIP for Further Study

**Figure 2:**
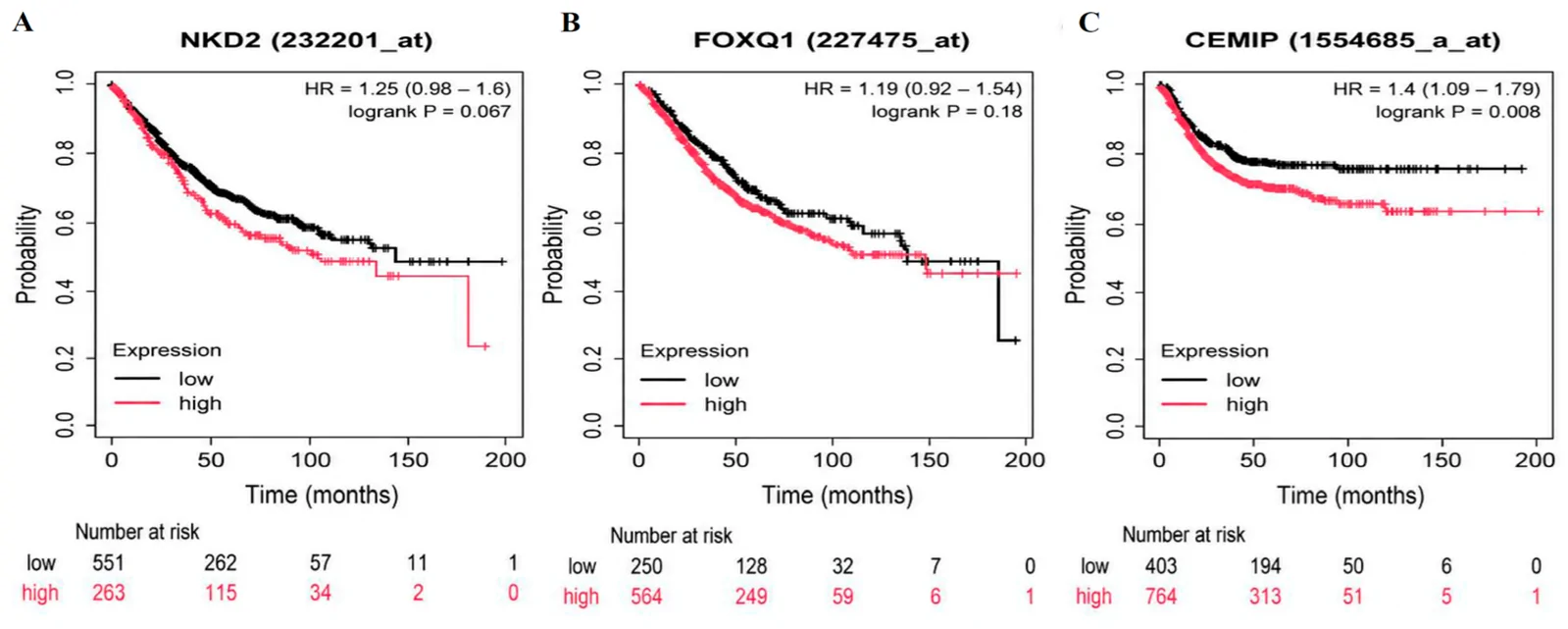
Overall survival analysis of NKD2, FOXQ1, and CEMIP in colorectal cancer. Kaplan–Meier curves compare high- and low-expression groups for NKD2 (**A**), FOXQ1 (**B**), and CEMIP (**C**). The number-at-risk tables beneath the curves report the numbers of patients remaining under observation in the low-expression (black) and high-expression (red) groups at 0, 50, 100, 150, and 200 months. Only CEMIP was significantly associated with poorer overall survival (HR = 1.40, 95% CI = 1.09–1.79; log-rank *p* = 0.008). These analyses were performed in an overall colorectal cancer cohort and were used for candidate prioritization rather than for EOCRC-specific prognostic inference.

To prioritize the three EOCRC-associated candidates, we evaluated their associations with overall survival in an independent colorectal cancer survival resource. High CEMIP expression was significantly associated with poorer survival (HR = 1.40, 95% CI = 1.09–1.79; log-rank *p* = 0.008), whereas NKD2 showed a borderline association (HR = 1.25, 95% CI = 0.98–1.60; *p* = 0.067) and FOXQ1 was not significant (HR = 1.19, 95% CI = 0.92–1.54; *p* = 0.18) (Figure 2). The numbers displayed beneath each Kaplan–Meier curve constitute the number-at-risk table and indicate how many patients in the low- and high-expression groups remained under observation at 0, 50, 100, 150, and 200 months. These overall CRC survival data were used for candidate prioritization and do not establish an EOCRC-specific prognostic effect. Clinicopathological and molecular subgroup analyses were not performed because complete individual-level covariates were not available through the survival interface. On the basis of its reproducible transcriptomic enrichment and significant overall survival association, CEMIP was selected for subsequent functional analyses.

### 3.3. CEMIP Is Highly Expressed in Selected CRC Cell Lines and Supports Viable Cell Biomass

**Figure 3:**
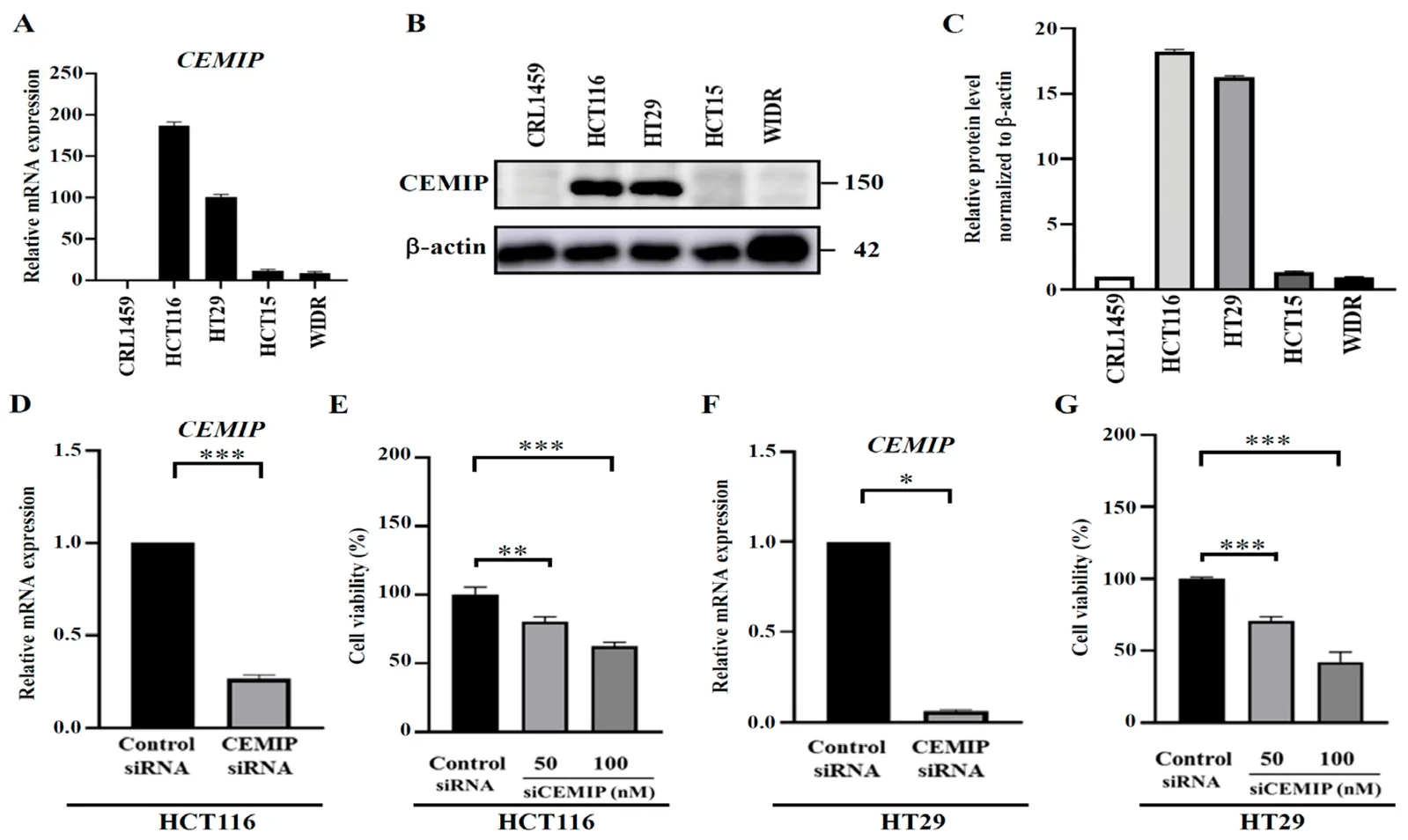
Basal CEMIP expression and effects of CEMIP knockdown on viable cell biomass. (**A**) Basal CEMIP mRNA expression in CCD-18Co, HCT116, HT29, HCT15, and WiDr cells. (**B**) Representative Western blot of basal CEMIP expression. (**C**) Densitometric quantification normalized to β-actin. (**D**,**F**) RT-qPCR confirmation of CEMIP mRNA knockdown in HCT116 and HT29 cells. (**E**,**G**) Crystal violet assays showing reduced relative viable cell biomass after treatment with 50 or 100 nM CEMIP siRNA. Panels D–G were horizontally aligned, and the CEMIP-targeting reagent is labeled consistently as CEMIP siRNA. Data are mean ± SD from three independent experiments. * *p* < 0.05; ** *p* < 0.01; *** *p* < 0.001.

We first examined the basal expression of CEMIP in the non-malignant colon-derived CCD-18Co cell line (ATCC CRL-1459) and four colorectal cancer cell lines, including HCT116, HT29, HCT15, and WiDr. RT-qPCR analysis showed marked variation in basal CEMIP mRNA expression among the tested cell models. HCT116 cells exhibited the highest CEMIP mRNA level, followed by HT29 cells, whereas substantially lower expression was detected in CCD-18Co, HCT15, and WiDr cells (Figure 3A). Consistent with the transcript-level findings, Western blot analysis demonstrated strong CEMIP expression in HCT116 and HT29 cells but only weak or minimal expression in the remaining cell lines (Figure 3B). Densitometric analysis normalized to β-actin confirmed the relatively high abundance of CEMIP in HCT116 and HT29 cells (Figure 3C). Based on these concordant mRNA and protein expression profiles, HCT116 and HT29 cells were selected for subsequent loss-of-function experiments. The efficiency of siRNA-mediated CEMIP silencing was subsequently evaluated by RT-qPCR. Relative CEMIP mRNA expression, normalized to 18S rRNA, was significantly reduced following CEMIP siRNA transfection in both HCT116 and HT29 cells compared with the corresponding control siRNA groups (Figure 3D,F). The reduction was pronounced in both models, with particularly strong suppression observed in HT29 cells. We next assessed the effect of CEMIP knockdown on viable adherent cell biomass using crystal violet staining. CEMIP silencing caused a significant and concentration-dependent reduction in relative viable cell biomass in both cell lines (Figure 3E,G). In HCT116 cells, treatment with 50 and 100 nM CEMIP siRNA reduced the crystal violet signal relative to control siRNA, with the greatest reduction observed at 100 nM. A similar but more pronounced dose-dependent response was observed in HT29 cells. These findings indicate that endogenous CEMIP contributes to the maintenance of viable adherent CRC cell biomass, although the assay does not distinguish among effects on proliferation, survival, and cell attachment.

### 3.4. CEMIP Knockdown Reduces the Migratory Capacity of CRC Cells

**Figure 4:**
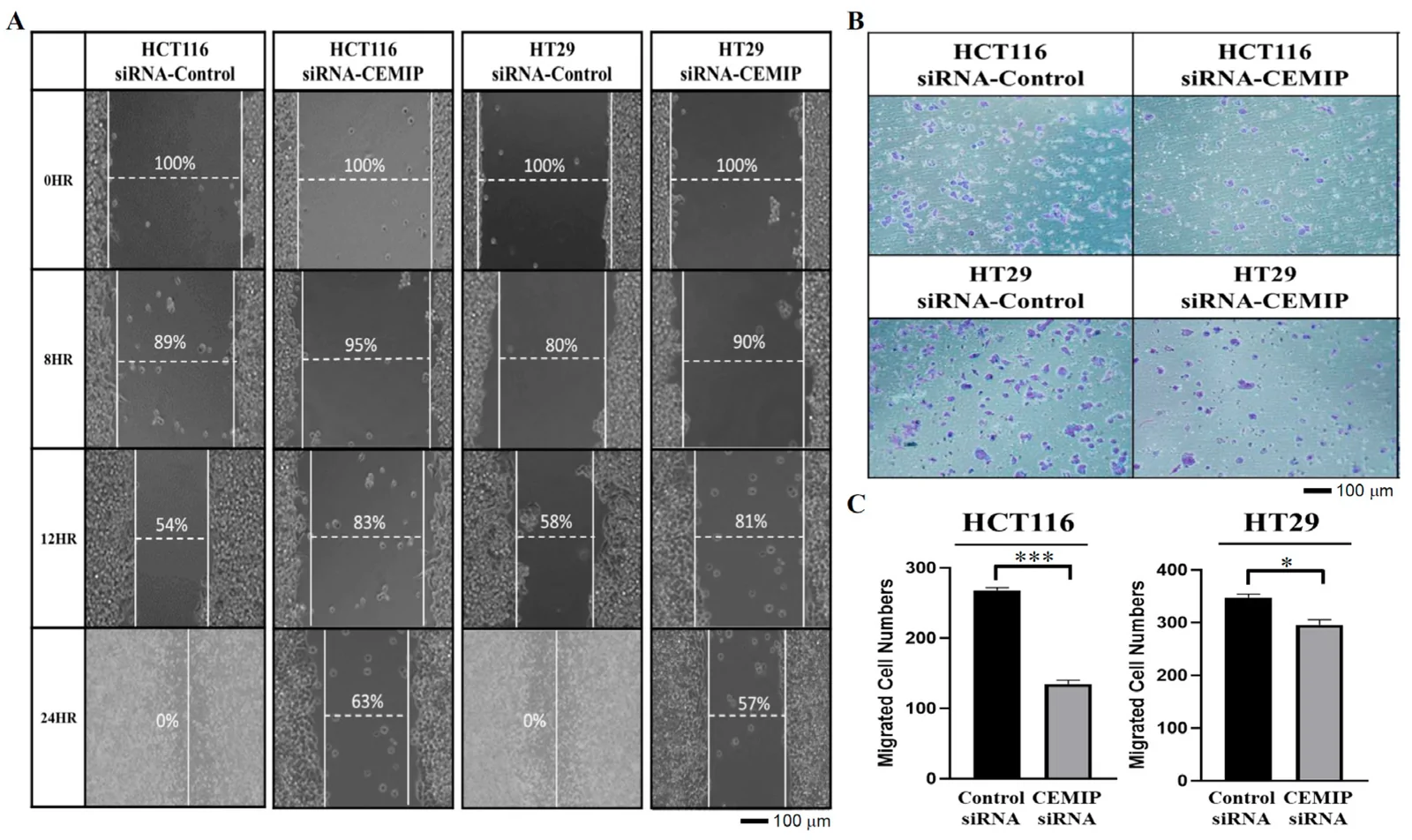
CEMIP knockdown reduces CRC cell migration. (**A**) Representative wound-healing images and wound-closure measurements in HCT116 and HT29 cells following transfection with control siRNA or CEMIP siRNA. (**B**) Representative Transwell migration images showing Giemsa-stained cells that migrated through the 8-μm-pore membrane and adhered to its lower surface. (**C**) Quantification of migrated cells. One representative scale bar is shown for each microscopy assay panel because all images within the corresponding panel were acquired at the same magnification. Scale bars = 100 μm. Data are presented as the mean ± SD from three independent experiments. The HCT116 comparison was significant at *** *p* < 0.001, whereas the HT29 comparison was significant at * *p* < 0.05.

We next investigated whether CEMIP contributes to CRC cell migration using complementary wound-healing and Transwell assays. In the wound-healing assay, the initial wound area was defined as 100% at 0 h for each experimental group. Control HCT116 cells showed progressive wound closure, with the remaining wound area decreasing to approximately 89% at 8 h and 54% at 12 h, followed by nearly complete closure at 24 h. In contrast, CEMIP-silenced HCT116 cells showed delayed wound closure, retaining approximately 95%, 83%, and 63% of the initial wound area at 8, 12, and 24 h, respectively (Figure 4A). A similar pattern was observed in HT29 cells. The remaining wound area in control cells decreased to approximately 80% at 8 h and 58% at 12 h, with nearly complete closure by 24 h. However, HT29 cells transfected with CEMIP siRNA retained approximately 90%, 81%, and 57% of the initial wound area at the corresponding time points (Figure 4A). These findings indicate that CEMIP knockdown consistently delays wound closure in both CRC cell models. The effect of CEMIP silencing on migration was further examined using the Transwell assay. Representative micrographs showed fewer migrated, Giemsa-stained cells after CEMIP knockdown in both HCT116 and HT29 cells compared with their respective control groups (Figure 4B). Quantification confirmed a significant reduction in migrated cell numbers following CEMIP silencing (Figure 4C). The reduction was particularly marked in HCT116 cells, in which the number of migrated cells was decreased by approximately one-half, whereas HT29 cells showed a smaller but statistically significant reduction. Taken together, the results from both wound-healing and Transwell assays consistently demonstrate that CEMIP knockdown impairs the migratory capacity of CRC cells. These data support a positive contribution of endogenous CEMIP to CRC cell motility, while the potential contribution of reduced viable cell biomass to the migration phenotype should also be considered.

### 3.5. CEMIP Expression Is Associated with PTCH1-Related Hedgehog Signaling

**Figure 5:**
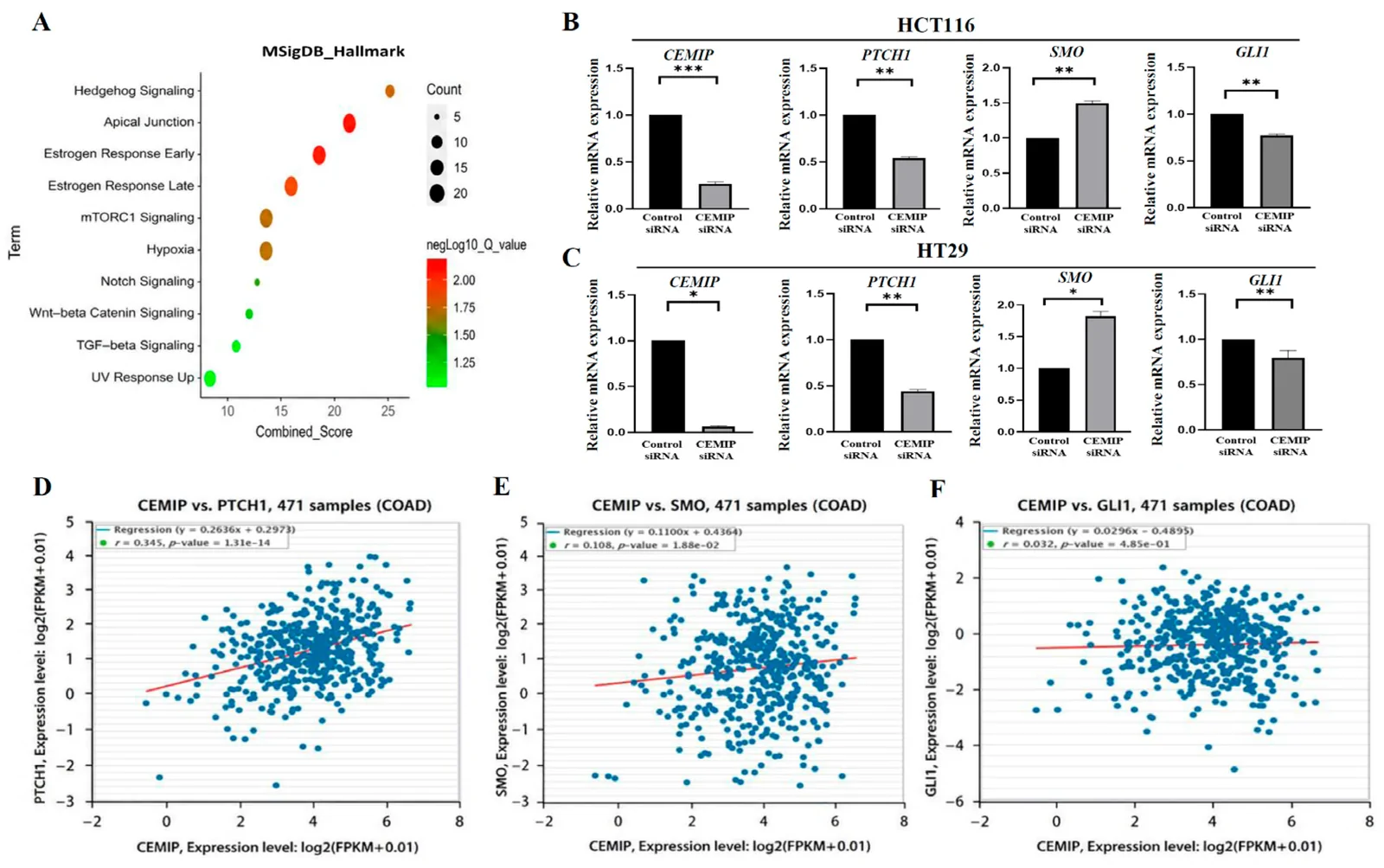
Exploratory association of CEMIP with PTCH1-related Hedgehog signaling. (**A**) MSigDB Hallmark 2020 enrichment analysis of genes associated with CEMIP overexpression in GSE39575. (**B**,**C**) RT-qPCR analysis of CEMIP, PTCH1, SMO, and GLI1 after CEMIP knockdown in HCT116 and HT29 cells; transcript levels were normalized to 18S rRNA and expressed relative to the corresponding control-siRNA group. (**D**–**F**) Redrawn correlation plots showing ENCORI associations of CEMIP with PTCH1 (r = 0.345, *p* = 1.31 × 10^−14^), SMO (r = 0.108, *p* = 1.88 × 10^−2^), and GLI1 (r = 0.032, *p* = 0.485) in TCGA-COAD samples. Axes report log_2_(FPKM + 0.01) expression values. The data support an association but not direct molecular interaction or causality. * *p* < 0.05; ** *p* < 0.01; *** *p* < 0.001.

To identify signaling pathways potentially associated with CEMIP, genes differentially expressed in CEMIP-overexpressing SW480 cells from the GSE39575 dataset were subjected to enrichment analysis using the MSigDB Hallmark gene sets. Hedgehog signaling was among the significantly enriched pathways, together with apical junction, estrogen-response, hypoxia, and mTORC1-related signatures. In contrast, Wnt/β-catenin and TGF-β signaling showed nominal enrichment but did not remain significant after adjustment for multiple comparisons (Figure 5A; Supplementary Table S1). We next examined the expression of selected Hedgehog-related genes after CEMIP knockdown in HCT116 and HT29 cells. The analyzed genes included Patched 1 (PTCH1), Smoothened (SMO), and GLI family zinc finger 1 (GLI1). Relative mRNA levels were normalized to 18S rRNA and expressed relative to the corresponding control siRNA group. In both cell lines, CEMIP silencing significantly reduced PTCH1 and GLI1 mRNA expression, whereas SMO mRNA expression was increased (Figure 5B,C). To further assess these relationships in clinical colorectal cancer samples, we analyzed TCGA colon adenocarcinoma (TCGA-COAD) RNA-sequencing data. Gene expression values were reported as log2-transformed FPKM values [log2(FPKM + 0.01)]. CEMIP expression showed a moderate positive correlation with PTCH1 (r = 0.345, *p* = 1.31 × 10^−14^), a weak positive correlation with SMO (r = 0.108, *p* = 1.88 × 10^−2^), and no significant correlation with GLI1 (r = 0.032, *p* = 0.485) (Figure 5D–F). Taken together, these findings support an exploratory association between CEMIP expression and PTCH1-related Hedgehog signaling. However, the current data do not establish a direct molecular interaction, pathway dependency, or a linear CEMIP–PTCH1–SMO–GLI1 signaling cascade.

## 4. Discussion

In this study, integrated age-stratified transcriptomic analyses identified CEMIP, NKD2, and FOXQ1 as candidate genes enriched in EOCRC cohorts. Overall CRC survival analysis prioritized CEMIP, and loss-of-function experiments showed that CEMIP supports viable cell biomass and migration in HCT116 and HT29 cells. Pathway enrichment and gene-expression analyses additionally revealed an exploratory association between CEMIP and PTCH1-related Hedgehog signaling. Importantly, the revised interpretation distinguishes functional evidence for CEMIP in the measured cellular phenotypes from correlative evidence regarding the Hedgehog pathway.

EOCRC is increasingly viewed as a heterogeneous clinical entity rather than simply CRC occurring at a younger age. Systematic and case–controlled studies have reported differences in presentation, histology, and molecular profiles between EOCRC and LOCRC [55,56,57]. Our discovery cohort was small and incompletely annotated, but the direction of CEMIP, NKD2, and FOXQ1 expressions were reproduced in an integrated set of independent GEO cohorts. The survival analysis was conducted in an overall CRC population and therefore served only to prioritize CEMIP; it does not prove that CEMIP has an age-specific prognostic effect.

The functional phenotype observed after CEMIP knockdown is consistent with established CRC biology. CEMIP/KIAA1199 is induced during colorectal neoplastic progression and is associated with poor outcome [58]. Mechanistically, CEMIP has been linked to glutamine metabolic reprogramming and β-catenin regulation [59], MHC-I internalization and immune evasion [60], Wnt/β-catenin/Snail-dependent EMT [61], and GRAF1–MIB1–CDC42/MAPK-mediated EMT and metastasis [62]. These pathways provide a stronger established mechanistic context for the reduced cell viability and migration observed here than the currently exploratory Hedgehog association.

Recent studies further broaden the relevance of CEMIP to stemness and inflammatory tumor ecology. The ELK4–METTL3–CEMIP axis was reported to enhance colorectal carcinoma cell growth, migration, invasion, spheroid formation, and stemness [63]. In addition, an inflammation-associated tumor-microenvironment analysis identified CEMIP-positive monocytes as an immunosuppressive subgroup linked to unfavorable anti-PD-1 response [64], complementing evidence that tumor-derived CEMIP can reduce MHC-I surface presentation [60]. These observations suggest that future EOCRC studies should evaluate CEMIP in tumor epithelial cells and immune/stromal compartments rather than assuming a tumor-cell-autonomous mechanism.

Our pathway analysis requires cautious interpretation. STRING does not support a direct CEMIP–PTCH1 protein interaction, and no complementary PTCH1 knockdown, rescue, ligand stimulation, or pathway-inhibition experiments were performed. Moreover, the correlation with PTCH1 was moderate, the correlation with SMO was weak, and GLI1 was not correlated in TCGA-COAD. Accordingly, the data support an association with PTCH1-related Hedgehog signaling rather than a direct mechanistic axis. The decrease in PTCH1 and GLI1 together with an increase in SMO after CEMIP silencing may reflect compensatory feedback, altered receptor trafficking, or non-canonical Hedgehog regulation. Because transcript abundance does not directly establish pathway activity, future studies should measure GLI-dependent transcription, protein localization, and pharmacologic or genetic rescue.

From a translational perspective, CEMIP may be useful as part of a molecular stratification panel for CRC, particularly if its age-associated expression is validated in larger clinically annotated EOCRC cohorts. Direct drug development against CEMIP remains challenging because it is a multifunctional extracellular and intracellular protein; however, its established links to CDC42/MAPK signaling, metabolic adaptation, stemness, and immune evasion suggest rational combination strategies. Potential approaches include testing CEMIP-directed agents or pathway inhibitors alongside immune-checkpoint blockade, metabolic therapies, or agents that target EMT/stemness programs. By contrast, combining CEMIP targeting with Hedgehog inhibitors would be premature until direct pathway dependence is demonstrated.

This study has several limitations. First, the EOCRC discovery group was small and imbalanced relative to the LOCRC group, and the available public metadata did not support comprehensive age-specific survival or clinicopathological subgroup analyses. Second, HCT116 and HT29 were selected as EOCRC-derived models according to the reported age of the patients from whom the tumors originated and because of their relatively high endogenous CEMIP expression; however, two established cell lines cannot reproduce the biological and clinical heterogeneity of EOCRC, and age at tumor derivation alone does not establish an EOCRC-specific cellular phenotype. Third, the functional experiments were limited to siRNA-mediated loss of function without sequence disclosure, protein-level knockdown confirmation, rescue experiments, patient-derived models, or in vivo validation. Finally, the PTCH1/Hedgehog findings are based on enrichment, transcript-level perturbation, and correlation analyses and should therefore remain hypothesis-generating.

Future investigations should validate CEMIP expression in independent EOCRC tissue cohorts with detailed TNM stage, grade, molecular subtype, treatment, and outcome data; incorporate patient-derived organoids and in vivo models; and test whether CEMIP perturbation alters EMT, inflammasome/inflammatory signaling, stemness, immune evasion, and Hedgehog pathway activity. Such studies will determine whether CEMIP is primarily a biomarker, a context-dependent functional contributor, or a tractable therapeutic target in EOCRC.

## 5. Conclusions

Integrated transcriptomic analyses identified CEMIP, NKD2, and FOXQ1 as candidate genes associated with age-defined EOCRC cohorts, and overall CRC survival analysis prioritized CEMIP for functional evaluation. CEMIP knockdown reduced relative viable cell biomass and migration in CRC cells. CEMIP also showed an exploratory association with PTCH1-related Hedgehog signaling; however, the available data do not establish a direct interaction or pathway-mediated causality. CEMIP should therefore be considered a candidate EOCRC biomarker and functional contributor whose mechanistic and translational relevance requires validation in clinically annotated cohorts and direct pathway-modulation models.

## Figures and Tables

**Table 1:** Early-onset and late-onset colon cancer details in CRC Clinic. (GSE21815, Japanese CRC patient profile).

Characteristic	EOCRC		LOCRC	
	Number	%	Number	%
Number of patients	14	100%	123	100%
Gender				
Male	10	71%	68	55%
Female	4	29%	55	45%
Age at diagnosis (years)				
31–40	4	29%	0	0
41–50	10	71%	0	0
51–60	0	0	35	28%
61–70	0	0	42	33%
71–80	0	0	38	22%
>80	0	0	8	7%
TNM stage				
1	0	0	12	10%
2	2	15%	25	20%
3A	0	0	11	9%
3B	0	0	5	4%
4	3	21%	8	7%
ND	9	64%	62	50%

## Data Availability

Publicly available gene-expression datasets were obtained from the NCBI Gene Expression Omnibus under accession numbers GSE21815, GSE39575, GSE20970, GSE36335, GSE37892, GSE45404, GSE56386, GSE73360, GSE89076, GSE101896, GSE106582, and GSE161158. Survival data were analyzed using Kaplan–Meier Plotter, and gene-correlation data were analyzed using ENCORI/starBase. The processed data underlying the figures are available from the corresponding authors upon reasonable request.

## Supplementary Materials

The following supporting information can be downloaded at: https://www.mdpi.com/article/10.3390/genes17080922/s1, Table S1: The MSigDB_Hallmark_2020_table analysis comes from the GSE39575 data set, and the correlation analysis with the CEMIP expression.

## Abbreviations

The following abbreviations are used in this manuscript:

EOCRC	Early-onset colorectal cancer
CEMIP	Cell migration-inducing protein
Hh	Hedgehog
PTCH1	Patched 1
