# Supplementary material for: Age-Stratified Transcriptomic Profiling Identifies CEMIP as a Candidate Biomarker in Early-Onset Colorectal Cancer and Reveals an Association with PTCH1-Related Hedgehog Signaling
Source: Genes (Basel). 2026 Aug 4;17(8):922. doi: 10.3390/genes17080922 (PMC13512092; doi:10.3390/genes17080922)
Supplement: Supplementary file 1 [file genes-17-00922-s001.zip › genes-4418952-supplementary.pdf]

Supplementary Materials

Table S1. The MSigDB_Hallmark_2020_table analysis comes from the GSE39575 data set, and the correlation analysis with the CEMIP expression

Term	Overlap	P_value	Adjusted
Apical Junction	24/200	0.000127891	6.39E-03
Estrogen Response Early	23/200	0.000327606	8.19E-03
Estrogen Response Late	22/200	0.000801297	1.34E-02
Hypoxia	21/200	0.00186886	1.87E-02
mTORC1 Signaling	21/200	0.00186886	0.018688595
Hedgehog Signaling	7/36	0.002295018	0.01912515
Mitotic Spindle	18/199	0.016775195	0.090263813
IL-2/STAT5 Signaling	18/199	0.016775195	9.03E-02
p53 Pathway	18/200	0.017567551	0.090263813
UV Response Up	15/158	0.018564287	9.03E-02
Wnt-beta Catenin Signaling	6/42	0.021045898	9.03E-02
TGF-beta Signaling	7/54	0.021663315	9.03E-02
Notch Signaling	5/32	0.024002152	9.23E-02
Myogenesis	17/200	0.033381486	0.119219594
PI3K/AKT/mTOR Signaling	10/105	0.047813661	0.159378869
Oxidative Phosphorylation	16/200	0.060035774	0.187611794
Unfolded Protein Response	10/113	0.071491788	0.206717297
UV Response Dn	12/144	0.074418227	0.206717297
Myc Targets V2	6/58	0.081259977	2.14E-01
Cholesterol Homeostasis	7/74	0.091251926	0.228129816
Adipogenesis	15/200	0.102047078	0.231925178
Glycolysis	15/200	0.102047078	0.231925178
Androgen Response	8/100	0.151903978	0.330226038
Xenobiotic Metabolism	14/200	0.163727958	0.341099912
Myc Targets V1	13/200	0.247712384	0.435513149
Epithelial Mesenchymal Transition	13/200	0.247712384	0.435513149
heme Metabolism	13/200	0.247712384	0.435513149
KRAS Signaling Up	13/200	0.247712384	0.435513149
Reactive Oxygen Species Pathway	4/49	0.252597626	0.435513149
Pperoxisome	7/104	0.300924183	0.501540305

Fatty Acid Metabolism	10/158	0.31251122	0.504050355
Apoptosis	10/161	0.333219981	0.519447732
Pancreas Beta Cells	3/40	0.348276457	0.519447732
E2F Targets	12/200	0.353224458	0.519447732
Protein Secretion	6/96	0.386863891	0.552662702
Apical Surface	3/44	0.405868207	0.563705843
G2-M Checkpoint	11/200	0.47481395	0.630952065
IL-6/JAK/STAT3 Signaling	5/87	0.47952357	0.630952065
DNA Repair	8/150	0.527770034	0.676628248
Spermatogenesis	7/135	0.562974808	0.692472559
Angiogenesis	2/36	0.567827498	0.692472559
TNF-alpha Signaling via NF-kB	10/200	0.602447995	0.700520925
Complement	10/200	0.602447995	0.700520925
KRAS Signaling Dn	9/200	0.723486049	0.822143238
Interferon Alpha Response	4/97	0.752661535	0.836290595
Interferon Gamma Response	8/200	0.826148052	0.860570888
Inflammatory Response	8/200	0.826148052	0.860570888
Allograft Rejection	8/200	0.826148052	0.860570888
Bile Acid Metabolism	3/112	0.936564341	0.955677899
Coagulation	3/138	0.977625887	0.977625887
